# Association Between *Ginkgo Biloba* Extract Prescriptions and Dementia Incidence in Outpatients with Mild Cognitive Impairment in Germany: A Retrospective Cohort Study

**DOI:** 10.3233/JAD-215348

**Published:** 2022-03-22

**Authors:** Jens Bohlken, Oliver Peters, Karel Kostev

**Affiliations:** a Institute for Social Medicine, Occupational Medicine, and Public Health (ISAP) of the Medical Faculty at the University of Leipzig, Leipzig, Germany; b Department of Psychiatry, Charité-Universitätsmedizin Berlin, Corporate Member of Freie Universität Berlin and Humboldt-Universität zu Berlin, Berlin, Germany; c German Center for Neurodegenerative Diseases (DZNE), Berlin, Germany; d Epidemiology, IQVIA, Frankfurt am Main, Germany

**Keywords:** Dementia, *Ginkgo biloba* extract, mild cognitive impairment, outpatients

## Abstract

**Background:**

Clinical trials have demonstrated a significant effectiveness of *Ginkgo biloba* therapy versus placebo in patients with dementia.

**Objective:**

The present study aims to analyze the impact of *Ginkgo biloba* drug prescriptions on dementia incidence in patients with mild cognitive impairment (MCI) in a real-world setting.

**Methods:**

This retrospective study was based on the IQVIA Disease Analyzer database and included patients aged 65 or older with a first diagnosis of MCI from January 2000 to December 2019. Each patient was followed for up to 20 years after MCI diagnosis until February 2021. Date of the first diagnosis of dementia or loss to follow-up, whichever occurred first, was noted. To estimate the association between *Ginkgo biloba* prescriptions during the follow-up and dementia incidence, a multivariable Cox regression analysis was performed, adjusted for age, sex, health insurance, documented co-diagnoses, and prescription of cholinesterase inhibitors.

**Results:**

Overall, 24,483 MCI patients (mean age: 77.0 years, 56.3% women) were included. It was found that > 2 prescriptions of *Ginkgo biloba* were significantly associated with a reduced dementia incidence (HR: 0.71 (95% CI: 0.55–0.91), *p* = 0.007), as compared with no *Ginkgo biloba* prescription. The effect of receiving > 3 *Ginkgo biloba* prescriptions was even stronger, with an HR of 0.64 (95% CI: 0.48–0.86), *p* = 0.003), while for > 4 prescriptions the HR was 0.58 (95% CI: 0.41–0.82) (*p* = 0.002).

**Conclusion:**

All-cause dementia incidence decreased with higher numbers of *Ginkgo biloba* prescriptions in MCI patients.

## INTRODUCTION

There are currently around 1.7 million people living with dementia in Germany, with more than 300,000 people newly diagnosed with the syndrome each year [1]. The concept of mild cognitive impairment (MCI) represents a state of cognitive function between that seen in normal aging and dementia [2]. A recent report comparing six European countries estimated that the prevalence of MCI in Germany was 3.7 million patients in 2019 [3]. In patients followed in general practices, the progression of MCI to dementia increased from 6.6% in the age group ≤60 years to 39.0% in the age group > 80 years [4]. Thus, it is important for health care providers to be aware of the condition and place it in the appropriate clinical context [5].

Several research teams studied the impact of *Ginkgo biloba* extract (Gbe), mainly special extract EGb 761, on the long-term risk of dementia in elderly people. A significant effect of Gbe was observed in a placebo-controlled pilot trial in elderly patients when compliance was considered as covariate [6]. No effect was observed in the placebo-controlled GEM trial with EGb 761 in healthy elderly patients [7]. However, low dementia incidence and low treatment compliance may skew the interpretation of this trial. A late effect of EGb 761 was observed in the placebo-controlled GUIDAGE trial in subjective memory impairment patients. However, the effect was not statistically significant in the primary analysis [8, 9]. A Chinese group also reported significantly reduced dementia incidence in amnestic MCI in a placebo-controlled 12-month trial with EGb 761 [10].

Key randomized trials and robust meta-analyses have demonstrated significant improvement in cognitive function, neuropsychiatric symptoms, activities of daily living, and quality of life with EGb 761 versus placebo in patients with mild-to-moderate dementia [11]. In those with MCI, EGb 761 was also found to cause significant symptomatic improvement versus a placebo [12, 14].

The previously published studies were placebo-controlled clinical trials. Few of the published studies used real world data in order to benefit from the combination of large patient numbers, long follow-up time, and controls for several co-variables. Dartigues et al. found, in 3,777 elderly people in France, that EGb 761 consumers had a lower risk of dying prior to a dementia diagnosis and a longer lifetime prior to a dementia diagnosis than participants taking other drugs for the same indication [13]. However, there are no studies based on large numbers of outpatients followed in GP or neuropsychiatrist practices. The present study aims to close the current gap in the research and literature by analyzing the association of Gbe prescriptions and dementia incidence in outpatients with MCI in a real-world setting.

## METHODS

### Data source

This analysis was based on the IQVIA Disease Analyzer database, which contains information on patient demographics, drug prescriptions, diagnoses, sick leave, and referrals to hospitals provided by general practitioners (GPs) and specialists in Germany. Information is provided by nearly 3,000 office-based physicians, representing approximately 3% of all German practices, covering eight major German regions.

IQVIA ensures the accuracy, consistency, and completeness of the data, and the database is suitable for pharmaco-epidemiological and pharmaco-economic studies [15]. Finally, this database has already been used in previous studies focusing on MCI and dementia [16–18].

### Ethics statement

German law allows the use of anonymous, de-identified electronic medical records for research purposes under certain conditions. According to German legislation, it is not necessary to obtain informed consent from patients or approval from a medical ethics committee for this type of observational study that contains no directly identifiable data. Therefore, no waiver of ethical approval can be obtained from an Institutional Review Board (IRB) or ethics committee. The funding company and the involved authors had no access to any identifying information at any moment during the analysis of the data.

### Study population

This retrospective database study included patients aged 65 or older in an outpatient care setting in Germany, including all patients in GP and neuropsychiatrist practices from the IQVIA Disease Analyzer database, with a first diagnosis of MCI (ICD-10: F06.7) from January 2000 to December 2019 (index date). Patients with a diagnosis of dementia (ICD-10: F00-F03, G30) prior to the index date were excluded.

### Study outcome

The main outcome of this study was the association between Gbe prescriptions and dementia incidence. Patients were subject to follow-up for up to 20 years after the index date. The last month for data retrieval was February 2021.

### Statistical analyses

The study population was descriptively characterized using age as a continuous variable, as well as sex, insurance status (private or statutory), co-diagnoses documented prior to the index date or during the follow-up time (hypertension (ICD-10: I10 + prescription of antihypertensive drug (ATC: C03, C07, C08, C09)), diabetes mellitus (ICD-10: E10–E14 + prescription of antihyperglycemic drug (ATC: C10)), stroke/TIA (ICD-10: I63, I64, G45), depression (ICD-10: F32, F33 plus prescription of antidepressant (ATC: N06A)), hearing impairment (ICD-10: H90, H91), traumatic brain injury (ICD-10: S07)), as well as prescription of cholinesterase inhibitor in the time between index date and prior to the date of dementia diagnosis (not including the date of dementia diagnosis) or end of follow-up (when no dementia diagnosis is present).

To estimate the association between Gbe prescriptions and dementia incidence, a multivariable Cox regression analysis was performed, adjusted for age, sex, health insurance, co-diagnoses documented until the end of follow-up, and prescription of cholinesterase inhibitors during the follow-up. Three different Cox regression models were used. In the first model, prescriptions of Gbe between the index date and the first dementia diagnosis or last visit (impact variables) were classified into two categories (1–3 prescriptions, > 3 prescriptions) and compared with a reference group of patients without Gbe prescriptions (Reference group = 0 prescriptions). In the second model, prescriptions of Gbe were classified into three categories (1–2, 3–4, > 4 prescriptions) compared with no prescriptions. In a third model, 1 prescription, 2 prescriptions, and > 2 prescriptions were compared to no prescriptions.

In addition, regression models were fitted separately for male and female patients, for three age groups (65–74, 75–84, and ≥85), and for patients with private and statutory health insurance coverage.

## RESULTS

### Baseline characteristics of study patients

Overall, 24,483 MCI patients were included in the study. The follow up-period was 3.8 years on average. The demographic and clinical characteristics of the study sample are displayed in Table 1. In total, 56.3% of the subjects were women, the mean (standard deviation) age at MCI diagnosis was 77.0 (6.3) years, and 9.3% were covered by private health insurance. Moreover, 27.3% had been diagnosed with hypertension, 8.9% with diabetes mellitus, 11.8% with depression, 7.4% with stroke/TIA, 5.1% with hearing impairment, and only 0.9% with traumatic brain injury. During the follow-up time, 3.0% received at least one Gbe prescription (83 of them ≥240 mg daily) and 3.1% had at least one prescription of a cholinesterase inhibitor. In patients who received Gbe prescription, the first prescription was given after a median time of 11 days, and 35% received it on the day of the MCI diagnosis.

**Table 1:** Demographic and clinical characteristics of the study patients

Variable	All patients	Patients without dementia	Patients with dementia	*p* ^*^
*N*	24,483	18,278	6,205
Follow-up time (y) (Mean, SD)	3.8 (2.4)	3.8 (2.4)	3.9 (2.2)	< 0.001
Age at MCI diagnosis (Mean, SD) (Min-Max)	77.0 (6.3)	76.8 (6.3)	77.7 (6.0)	< 0.001
	(65–97)	(65–97)	(65–96)
Age 65–74	35.7	37.3	30.9	< 0.001
Age 75–84	51.4	50.3	54.6
Age≥85	12.9	12.4	14.5
Female	56.3	55.8	57.9	0.005
Male	43.7	44.2	42.1
Statutory health insurance	90.7	90.1	92.6	< 0.001
Private health insurance	9.3	9.9	7.4
Co-diagnoses documented prior to the end of follow-up (%)
Hypertension	27.3	28.5	23.8	< 0.001
Diabetes mellitus	8.9	9.7	6.8	< 0.001
Stroke/TIA	7.4	7.5	7.1	0.306
Depression	11.8	12.0	11.3	0.156
Hearing impairment	5.1	5.1	5.3	0.553
Traumatic brain injury	0.9	0.9	1.0	0.791
Gbe prescription
≥1 prescription	3.0	2.6	4.1	< 0.001
≥2 prescriptions	1.3	1.1	1.6	0.006
≥3 prescriptions	0.8	0.8	1.0	0.050
≥4 prescriptions	0.6	0.6	0.7	0.189
At least one prescription of cholinesterase inhibitor	3.1	2.3	5.7	< 0.001

^*^Baseline characteristics were compared in patients with and without dementia using chi-squared tests for categorical variables and Wilcoxon tests for continuous variables.

In total, 6,205 of 24,483 MCI patients (25.3%) had a dementia diagnosis. Patients with dementia during follow-up were slightly older at MCI diagnosis (77.7 versus 76.8 years), with a slightly higher proportion of females (57.9% versus 55.8%), lower proportion of private health insurance holders (7.4% versus 9.9%), as well as lower proportions of individuals diagnosed with hypertension (23.8% versus 28.5%) and diabetes (6.8% versus 9.7%) (Table 1).

### Association between Gbe prescriptions and dementia incidence

**Fig. 1:**
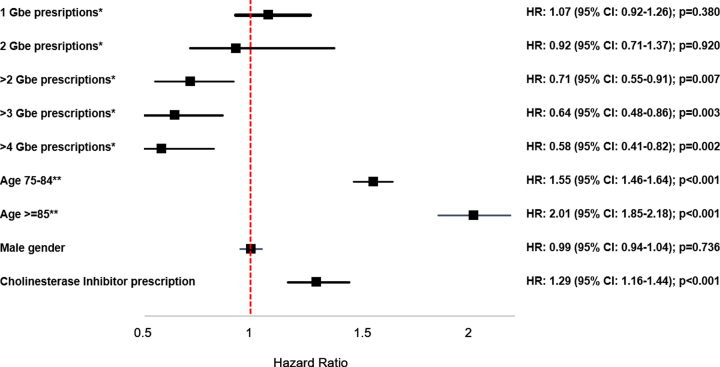
Association between *Ginkgo biloba* extract prescriptions and the incidence of dementia. Cox regression model adjusted for age, sex, health insurance status coverage, co-diagnoses documented prior to dementia diagnosis or end of follow-up (hypertension, diabetes mellitus, depression, hearing impairment, traumatic brain injury, stroke/TIA), and cholinesterase inhibitor prescription. ^*^Reference group: no Gbe prescriptions; ^**^Reference group: 65–74 years.

Figure 1 shows the results of the multivariable regression models, which found that > 2 prescriptions of Gbe were significantly associated with a reduced dementia incidence (HR: 0.71 (95% CI: 0.55–0.91), *p* = 0.007) as compared with no Gbe prescription. The effect of receiving > 3 Gbe prescriptions was even stronger, with an HR of 0.64 (95% CI: 0.48–0.86), *p* = 0.003), while for > 4 prescriptions the HR was 0.58 (95% CI: 0.41–0.82) (*p* = 0.002).

The effects of the co-variables were equal in all three models, with the strongest effect for age (HR: 1.55 (95% CI: 1.46–1.64) for age group 75–84 years, and HR: 2.01 (95% CI: 1.85–2.18) for age group≥85 compared to age group 65–74 years). Persons who received at least one prescription of a cholinesterase inhibitor had a higher dementia incidence (HR: 1.29 (95% CI: 1.16–1.44)). Persons with hypertension had a slightly reduced dementia risk (HR: 0.92 (95% CI: 0.82–1.02)), whereas diabetes mellitus had no effect (HR: 1.04 (95% CI: 0.80–1.34)), and persons suffering from depression (HR: 1.15 (95% CI: 1.05–1.27)) had an increased risk of dementia.

Table 2 shows the association between Gbe prescriptions and dementia incidence in defined subgroups. The strength of the association increased with age and was stronger in men than in women, as well as in privately insured patients compared to statutorily insured patients (Table 2).

**Table 2:** Association between Gbe prescriptions and the incidence of dementia in patient subgroups (Multivariable Cox regression model)

Variable	Adjusted Hazard Ratio (95% CI) for > 2 Gbe prescriptions versus no Gbe prescriptions	*p*	Adjusted Hazard Ratio (95% CI) for > 3 Gbe prescriptions versus no Gbe prescriptions	*p*	Adjusted Hazard Ratio (95% CI) for > 4 Gbe prescriptions versus no Gbe prescriptions	*p*
Age 65–74^*^	0.91 (0.61–1.36)	0.650	0.74 (0.46–1.19)	0.213	0.59 (0.33–1.05)	0.071
Age 75–84^*^	0.63 (0.44–0.90)	0.011	0.59 (0.38–0.89)	0.013	0.56 (0.34–0.91)	0.021
Age≥85^*^	0.56 (0.27–1.19)	0.131	0.59 (0.26–1.33)	0.0204	0.57 (0.21–1.53)	0.265
Female^**^	0.75 (0.55–1.02)	0.070	0.69 (0.48–0.98)	0.040	0.68 (0.45–1.03)	0.066
Male^**^	0.64 (0.42–0.98)	0.040	0.55 (0.33–0.93)	0.027	0.42 (0.22–0.80)	0.009
Statutory health insurance^***^	0.79 (0.60–1.04)	0.094	0.68 (0.48–0.95)	0.023	0.60 (0.40–0.90)	0.012
Private health insurance^***^	0.38 (0.21–0.67)	0.001	0.41 (0.22–0.75)	0.004	0.35 (0.17–0.71)	0.004

Age means age at time of MCI diagnosis. ^*^Cox regression model adjusted for sex, health insurance status coverage, co-diagnoses documented prior to dementia diagnosis or end of follow-up (hypertension, diabetes mellitus, depression, hearing impairment, traumatic brain injury, stroke/TIA), and cholinesterase inhibitor prescription. ^**^Cox regression model adjusted for age, health insurance status coverage, co-diagnoses documented prior to dementia diagnosis or end of follow-up (hypertension, diabetes mellitus, depression, hearing impairment, traumatic brain injury, stroke/TIA), and cholinesterase inhibitor prescription. ^***^Cox regression model adjusted for age, sex, co-diagnoses documented prior to dementia diagnosis or end of follow-up (hypertension, diabetes mellitus, depression, hearing impairment, traumatic brain injury, stroke/TIA), and cholinesterase inhibitor prescription.

## DISCUSSION

In this large retrospective cohort study, prescriptions of Gbe were associated with a significantly lower incidence of dementia in MCI patients followed for an average of 3.8 years and a maximum of up to 20 years. The incidence of dementia was statistically significantly reduced for patients who had received at least 3 prescriptions of Gbe. The strongest association was observed in patients with more than four prescriptions. These associations were adjusted for age, sex, health insurance status, as well as other diagnoses associated with dementia [19].

The possible mechanisms of action of Gbe include anti-oxidation and anti-inflammation [20], both of which are considered to contribute to cognitive improvement. As a consequence, Gbe could protect against dementia, in particular in MCI patients [21]. Le Bars et al. have shown that in mild forms of dementia, daily administration of 120 mg of ginkgo extract EGb 761 improves cognitive performance and social skills [22]. It is possible that a minimum dose of 120 mg daily is necessary for a sufficient effect. Yuan et al. performed an overview of systematic reviews and reported that Gbe had potentially beneficial effects on cognitive performance, as well as on clinical global impression in the treatment of dementia when patients were treated for at least 22 weeks and received doses greater than 200 mg per day [23]. In the database used in the present study, no details on the daily dosage recommendation (i.e., once or twice daily) were available. However, 83% of patients who received a prescription of Gbe had a dosage of at least 240 mg per day. The dose–response effect is well known from the medical research and refers to the relationship between the dose of treatment and the probability of improvement, whereby both the daily dose and therapy length, as well as the interaction between the daily dose and therapy length, can be important [24].

The present study has a long follow-up time, with an average of 3.6 years and a maximum of 20 years. In the 20-year follow-up, population-based PAQUID study involving 3,777 patients, users of Gbe were compared with users of nootropic drugs such as piracetam or subjects taking no such preparation. Intake of Gbe was associated with significantly slower progression of cognitive impairment and a longer lifetime without dementia [13, 25].

Interestingly, in the randomized, double-blind, placebo-controlled clinical trial of Vellas et al., long-term (for a duration of five years) use of *Ginkgo biloba* extract EGb 761 (240 mg per day) did not significantly reduce the risk of progression to Alzheimer’s disease compared with placebo in the prespecified proportional hazards analysis [9]. However, the risk was not proportional over time. Taking into account a time-dependent effect, the HR was 0.51 [0.29–0.90] (*p* = 0.021) for a treatment period of≥3 years. Similar trends were observed when the cut-point was set at 2 or 4 years, as well as for the outcome Alzheimer’s disease or mixed dementia [9]. This indicates that an effect of Gbe on dementia incidence can only be expected with sufficient treatment duration. In a Chinese placebo-controlled trial, an effect was only seen from 36 weeks of treatment onwards. These results from placebo-controlled trials are in line with our observation of a dose–response effect.

The following general conditions of outpatient care in Germany should be considered, as they may influence the frequency of repeated prescriptions.

On the part of the prescribing physicians, it is of importance that there is currently no guideline recommendation for any drug treatment of MCI in Germany [26]. However, the approval status of Gbe since 2015 includes the possibility of a medical prescription for “age-associated cognitive impairment,” in addition to the possibility of purchase as an OTC medication. The MCI indication does not include any cost coverage by the statutory health insurance, but the drug is covered by most private health insurances. It can therefore be assumed that the prescription costs were covered by the patients themselves within the statutory health insurance category. The frequency of repeat prescriptions is thus probably influenced not only by the attitude of the prescribing physicians towards herbal medication, but also by their assessment of the importance of treating memory disorders.

On the patient side, it should be considered that the care needs of memory disorders are often not sufficiently taken into account [27]. This may be why patients bear the prescription costs themselves. For example, a recent, albeit small, study demonstrates that more than half of surveyed patients with MCI prefer herbal medications [28]. It is not known to what extent this corresponds to a general attitude or whether this is also due to the better media representation of the possibilities of OTC drugs such as Gbe compared to prescription drugs. In Germany, multiple prescription of Gbe is subject to special conditions that differ from the continuous prescription of antidementia drugs for dementia [29].

The two major strengths of this study are the length of the follow-up period and the large sample size. However, the study results should be interpreted in light of several limitations. First, assessments rely on ICD codes entered by GPs and neuropsychiatrists, and diagnosis codes do not allow for the separation of severity stages. MCI seems to be underdiagnosed in Germany, and the miscoding of MCI as dementia cannot be ruled out. Second, no psychological test results and no radiology results are available. Third, in order to buy herbal medicines that are sold over the counter, patients do not need a prescription from physicians. The database does not include data on the use of herbal medicines that patients buy without prescriptions. This can cause an underestimation of the effect of Gbe on dementia risk. Fourth, the database used show the prescription of the drug, but not the compliance of the therapy, i.e., if and how often or how long patients took the prescribed medication.

Fifth, data on lifestyle-related risk factors (smoking, alcohol, physical activity) are not available. Fifth, no educational effects in the form of school-leaving qualifications or years of education can be taken into account, although these could help identify patients who are particularly attentive, very health-conscious and financially strong, who used Gbe over a longer period of time. Finally, patients could only be observed in a single practice; when they received a diagnosis or prescription by another physician, such prescriptions could not be analyzed, as data from different practices cannot be linked.

In conclusion, all-cause dementia incidence was reduced in MCI outpatients who received at least 3 Gbe prescriptions and further decreased when Gbe was prescribed more than three and more than four times.
